# VDR Gene variation and insulin resistance related diseases

**DOI:** 10.1186/s12944-017-0477-7

**Published:** 2017-08-19

**Authors:** Fei-fei Han, Ya-li Lv, Li-li Gong, He Liu, Zi-rui Wan, Li-hong Liu

**Affiliations:** Beijing Chao-Yang Hospital, Capital Medical University, Beijing, 100020 China

**Keywords:** VDR Gene polymorphisms, Type 2 diabetes (T2DM), Metabolic syndrome (MetS), Polycystic ovarian syndrome (PCOS)

## Abstract

**Background:**

Vitamin D status may influence the risk of Insulin resistance related diseases such as Type 2 diabetes (T2DM), metabolic syndrome (MetS), and polycystic ovarian syndrome (PCOS). Several studies have assessed vitamin D receptor (VDR) gene polymorphism in relationship with these diseases; however, results remain inconsistent. Our study was conducted to elucidate whether VDR Gene polymorphisms could predict insulin resistance on a large scale.

**Methods:**

A meta-analysis using MEDLINE and EMBASE, was performed up to December 16th, 2016. Studies reporting association of vitamin D gene polymorphism with incident T2DM, MetS and PCOS outcomes were included and sub-group analysis by pigment of skin and latitude were performed.

**Results:**

A total of 28 articles based on four gene variation, and comprising 9232 participants with 5193 Insulin resistance related diseases patients were included. No significant associations of the VDR ApaI, BsmI, FokI and TaqI variant with Insulin resistance related diseases were found. However, sub-group analysis analysis showed that PCOS in TaqI (OR = 1.47, 95% CI = 1.03–2.09, *P* = 0.03) for T allele and MetS for G allele (OR = 1.41, 95% CI = 1.07–1.85, *P* = 0.01) in BsmI was significant association with VDR gene polymorphism. Simultaneously, sub-group analysis showed VDR ApaI rs7975232(G > T)variant was associated with insulin resistance related diseases in Asians (GG/GT + TT) (OR, 1.62; 95% CI, 1.03–2.53; *P* = 0.04) and population who lived in middle latitude district (30–60°) (GG/GT + TT) (OR, 1.22; 95% CI, 1.04–1.43; *P* = 0.02), VDR BsmI rs1544410 (A > G)and VDR Taq1rs731236 (T/C) variant were associated with insulin resistance related diseases in Caucasian (dark-pigmented).

**Conclusion:**

The results suggested that the association between insulin resistance related diseases and VDR ApaI, BsmI, FokI variant was more obvious in dark-pigmented Caucasians and Asians but not in Caucasian with white skin.

## Background

Vitamin D deficiency as a common health problem is a global problem, thought to be related to lack of sunlight exposure, and usually accompanied by reduced dietary intake [1]. The Vitamin-D receptor (VDR) was studied as a genetic factor of spine pathologies and plays a part in normal bone mineralization and remodeling. It is an endocrine member belongs to the nuclear receptor superfamily for steroid hormones. Its gene polymorphisms are thought to contribute to osteoarthritis, osteoporosis and degenerative disc disease. Also researchers found that VDR regulates vitamin D levels and calcium metabolism in the body and these are known to be associated with endocrine dysfunctions, insulin resistance [2, 3]. Vitamin D has been reported to influence glucose regulation via effects on insulin secretion and action [4]. Evidence is accumulating to suggest that altered vitamin D and Ca homoeostasis may play a role in the development of metabolic disturbances in insulin resistance related diseases [5–7]. More and more studies found that the vitamin D was useful for insulin resistance diseases [8–10].

T2DM, MetS, and IFG are common metabolic disorders which are observed with increasing prevalence, and which are caused by a complex interplay between genetic and environmental factors, and these metabolic disorders are all characterized by insulin resistance [11–13]. PCOS is by far the most common cause of anovulatory infertility and has been reported to be associated with insulin resistance (IR), hyperinsulinemia, dyslipidemia, and central obesity, which are all risk factors for the MetS, T2DM, and cardiovascular disease. Several studies have assessed vitamin D receptor gene polymorphism in relationship with these diseases; however, results remain inconsistent.

Vitamin D condition depends mainly on the sunlight and skin. It is both an environmental and biological determinant of health. Skin pigmentation may predispose subpopulations to vitamin D deficiency [14]. Some studies demonstrate that vitamin D deficiency is much higher in dark-pigmented population and Asians due to a reduced ability to produce vitamin D in their skin [15, 16]. Wondering whether there was any correlation or diverseness among these different population and their living latitude, in this research we also performed sub-group studies by skin pigmentation and latitude. Our study was conducted to elucidate whether VDR Gene polymorphisms could predict insulin resistance on a large scale.

## Methods

### Search strategy and selection criteria

Two investigators (Fei-fei Han, Ya-li Lv) independently searched PubMed and Embase (from 1980 until December 16th, 2016) database using the terms ((Gene polymorphism or gene variation)) AND (((((((diabetes mellitus) OR Diabetes) OR insulin resistance) OR metabolic syndrome) OR polycystic ovarian syndrome)) AND (vitamin D receptor OR VDR)).

Furthermore, we reviewed citations in the retrieved articles to search for additional relevant studies. Articles included in meta-analysis were in English or Chinese, with human subjects, published in primary literature and with no obvious overlap of subjects with other studies. The retrieved literatures were then read in their entirety to assess their appropriateness for the inclusion in this meta-analysis. Conference abstracts, case reports, editorials, review articles, and letters were excluded. We defined strict criteria for inclusion of studies. Studies were included if the exposure of interest was the VDR genotype.

### Data extraction

Two independent authors extracted data and reached a consensus on the author, year of publication, ethnicity, number of patients and controls and disease types.

### Statistical analysis

All statistical analyses were performed using Review Manager (Review Manager 5.0 software) and Stata/MP 11.0. Cochran’s w^2^ test and the inconsistency index (I^2^) were used to evaluate heterogeneity across the included studies. Random-effects model was applied in all the analysis. OR and their corresponding 95% confidence intervals (CI) were estimated. *Z*-test was performed to determine the statistical significance of pooled OR, and was considered significant when *P* < 0.05. We assessed potential publication bias by using a funnel plot and Egger’s test. Sensitivity analysis was performed by sequential removal (statistics of study remove) of individual studies (we did not show these results) [17].

## Results

### Eligible studies for meta-analysis

**Fig. 1:**
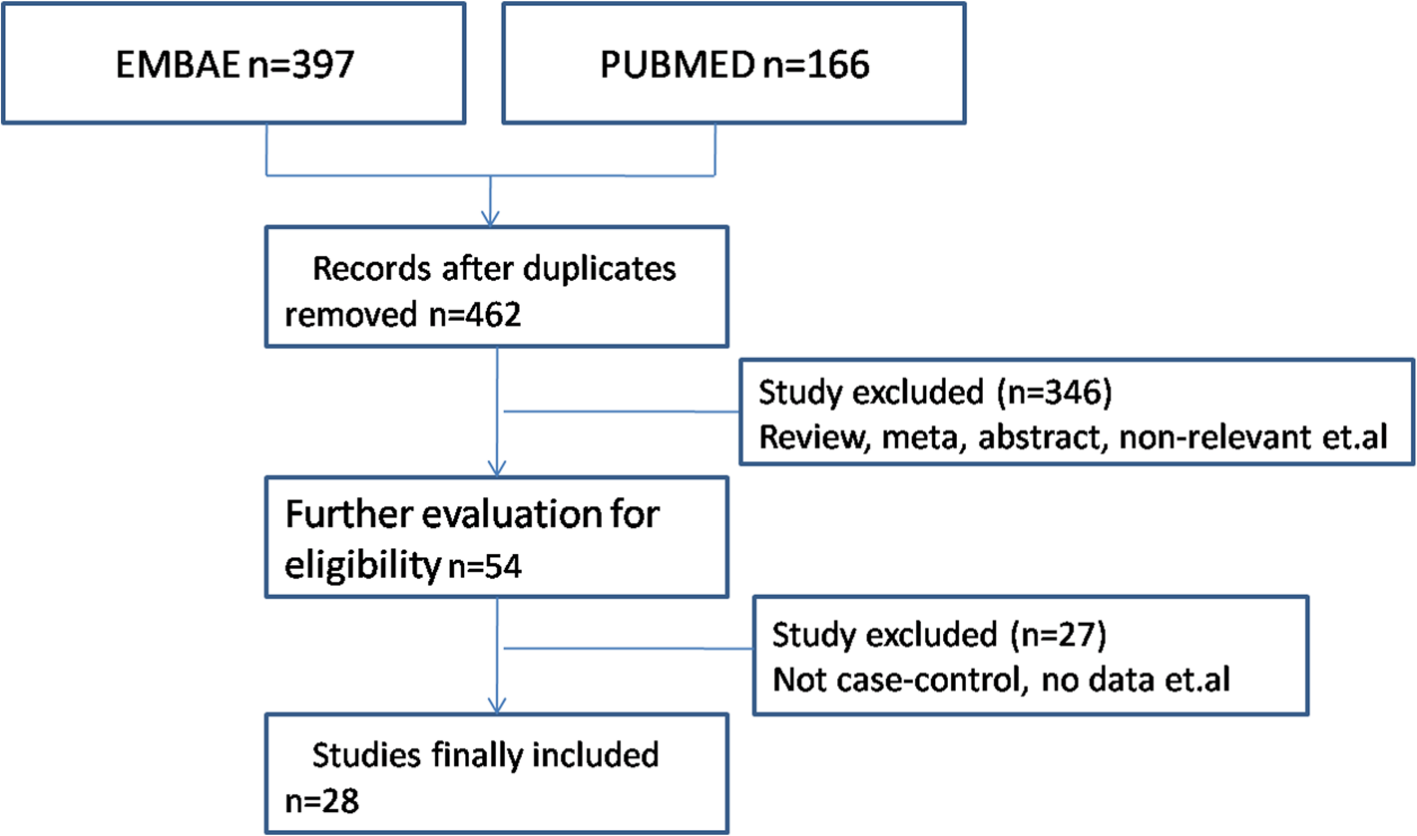
Flow diagram for study selection in meta-analysis

This study is focusing on VDR ApaI rs7975232 (G > T) variant, BsmI rs1544410 (A > G) variant, Taq1rs731236 (T > C) variant and FokIrs2228570 (C > T) variant and Insulin resistance related diseases susceptibility including (T2DM, MetS and PCOS). Characteristics of studies investigating the association of the variants with Insulin resistance related diseases susceptibility are presented in Table 1. The research of the VDR variant identified 54 articles. However, 26 studies were excluded for no case–control or no data. Finally, 28 studies were included in the current meta-analysis (Fig. 1).

**Table 1:** Characteristics of studies on VDR ApaI rs7975232 (G > T) variant and Insulin resistance related diseases susceptibility

Author	Year	Country	Ethic	City latitude	Disease	Case			Control		
						TT	TG	GG	TT	TG	GG
Al-Daghri NM [18]	2012	Saudi	Caucasian (dark)	Riyadh 24°38′N	T2DM	148	172	48	101	106	52
Boullu-Sanchis, S [19]	1999	France (migrant Indian population)	Caucasian (Dark)	Guadeloupe 16°15′N	T2DM	22	42	25	22	47	31
Dasgupta S [48]	2015	India	Caucasian (Dark)	Hyderabad 17°23′N	PCOS	117	120	13	120	117	13
Dilmec F [21]	2008	India	Caucasian (Dark)	Sanliurfa 37°17′N	T2DM	27	38	7	61	82	26
El-Shal AS [20]	2013	Egypt	Caucasian (Dark)	Zagazig 30°35′N	PCOS	63	65	22	68	64	18
Oh, J° Y° [22]	2001	USA	Caucasian	Southern California 32°42′N	T2DM	84	92	66	452	552	265
Jedrzejuk D [23]	2015	Poland	Caucasian	Wroclaw 51°1′N	PCOS	19	52	19	32	49	17
Mahmoudi T [24]	2009	Iran	Caucasian (Dark)	Tehran 35°40′N	PCOS	58	68	36	49	90	23
Malecki MT [25]	2003	Poland	Caucasian	Krakow 50°08′N	T2DM	71	153	84	60	124	56
Rivera-Leon EA [49]	2015	Mexico	Mix	Western of Mexico (Guadalajara 20°67′N)	T2DM	47	64	14	31	78	16
Wehr E [27]	2011	Austria	Caucasian	Graz 47°4′N	PCOS	142	274	127	48	60	37
Ye WZ [28]	2001	France	Caucasian	Paris 48°52′N	T2DM	98	142	65	35	78	30
Zhong X [30]	2015	China	Asian	Anhui Province 31°52′N	T2DM	29	114	61	28	59	29
Zhang H [29]	2012	China	Asian	Changsha 28°12′N	T2DM	30	154	120	12	53	35

Of these, 14 case–control studies examined the association of the ApaI rs7975232 (G > T) variant [3, 18–30] (Table 1), 22 studies in 20 case–control papers examined the association of the BsmI rs1544410 (A > G) variant [18, 22, 23, 27–39] (Table 2), 19 studies in 18 case–control studies examined the association of the Taq1rs731236 (T > C) variant[3, 18–28, 32, 33, 35, 38–40] (Table 3) and 18 studies in 16 case–control studies in15 papers examined the association of FokIrs2228570 (C > T)variant [3, 18, 23–25, 27, 30–32, 36, 41–45] (Table 4) with Insulin resistance related diseases susceptibility.

**Table 2:** Characteristics of studies on VDR BsmI rs1544410 (A > G) variant and Insulin resistance related diseases susceptibility

Author	Year	Country	Ethic	City latitude	Disease	Case			Control		
						GG	AG	AA	GG	AG	AA
Al-Daghri NM [18]	2012	Saudi	*Caucasian (dark)*	*Riyadh* 24°38′N	T2DM	105	201	62	114	95	50
Bagheri M [31]	2012	Iran	*Caucasian (dark)*	Urmia 37°33′N	PCOS	15	27	4	20	24	2
Bid HK [32]	2009	India	*Caucasian* *(dark)*	North Indian About 22–37°N	T2DM	30	52	18	60	77	23
Jedrzejuk D [23]	2015	Poland	*Caucasian*	Wroclaw 51°1′N	PCOS	31	45	14	43	42	13
Oh, J° Y° [22]	2001	USA	*Caucasian*	Southern California 32°42′N	T2DM	86	107	49	460	590	253
Mahmoudi T [24]	2009	Iran	*Caucasian (dark)*	Tehran 35°40′N	PCOS	53	85	24	53	91	18
Malecki MT [25]	2003	Poland	*Caucasian*	Krakow 50°08′N	T2DM	131	142	35	92	116	32
Mukhopadhyaya PN [33]	2010	India	*Caucasian (dark)*	Pune 18°52′N	T2DM	17	14	9	26	4	10
Mackawy A M [50]	2014	Eygpt	*Caucasian (dark)*	Zagazig 30°35′N	T2DM	17	33	80	9	16	38
					Mets	8	17	42	9	16	38
Speer G [34]	2001	Hungary	*Caucasian*	Budapest 47°30′N	MetS	40	46	14	33	48	19
Schuch NJ [42]	2013	Brazil	Mix	São Paulo 23°33′N	Mets	20	43	37	9	41	50
Vural HC [35]	2012	Turkey	*Caucasian*	Konya 37°86′N	T2DM	37	43	20	50	41	9
Wehr E [27]	2011	Austria	*Caucasian*	Graz 47°4′ N	PCOS	216	244	77	49	66	22
Xia Z [36]	2014	China	Asian	Beijing 39°26′-41°03′N	T2DM	209	27	2	82	8	1
Xu, J° R°[39]	2014	China	Asian	Ningxia province 35–39′N	T2DM	176	24	1	172	47	0
		Chinese hui population			T2DM	122	30	3	87	28	0
Xu JR [37]	2007	China	Asian	Ningxia province 35–39°N	T2DM	41	46	19	68	28	6
Ye WZ [28]	2001	France	*Caucasian*	Paris 48°52′N	T2DM	119	135	52	54	65	24
Zhang H [29]	2012	China	Asian	Changsha 28°12’N	T2DM	218	83	3	85	14	1
Zhong X [30]	2015	China	Asian	Anhui Province 31°52′N	T2DM	11	54	139	2	18	96
Yi Zhao [45]	2014	China	Asian	Yinchuan, Ningxia 38°2′N	MetS	347	42	1	328	69	3

**Table 3:** Characteristics of studies on VDR Taq1rs731236 (T/C) variant and Insulin resistance related diseases susceptibility

Author	Year	Ethic	Ethic	City latitude	Disease	Case			Control		
						CC	CT	TT	CC	CT	TT
Al-Daghri NM [18]	2012	Saudi	*Caucasian (dark)*	Riyadh 24°38′N	T2DM	65	195	108	50	114	95
Bagheri M [40]	2013	Iran	*Caucasian (dark)*	Urmia 37°33′N	PCOS	8	14	16	2	19	17
Bid HK [32]	2009	Indian	*Caucasian (dark)*	North Indian About 22–37°N	T2DM	15	49	36	28	65	67
Boullu-Sanchis, S [19]	1999	France	*Caucasian (dark)*	Guadeloupe 16°15′N	T2DM	48	33	8	44	39	17
Dasgupta S [48]	2015	India	*Caucasian (dark)*	Hyderabad 17°23′N	PCOS	47	92	113	37	105	110
Dilmec F [21]	2008	Turkey	*Caucasian*	Sanliurfa 37°17′N	T2DM	14	25	33	19	81	69
El-Shal AS [20]	2013	Egypt	*Caucasian (dark)*	Zagazig 30°35′N	PCOS	36	74	40	20	61	69
Oh, J° Y° [22]	2002	USA	*Caucasian*	Southern California 32°42′N	T2DM	41	108	93	219	581	503
Jedrzejuk D [23]	2015	Poland	*Caucasian*	Wroclaw 51°1′N	PCOS	8	45	37	12	37	49
Mahmoudi T [24]	2009	Iran	*Caucasian (dark)*	Tehran35°40′N	PCOS	20	71	71	14	76	72
Malecki MT [25]	2003	Poland	*Caucasian*	Krakow 50°08′N	T2DM	71	153	84	60	124	56
Mukhopadhyaya PN [33]	2010	Indian	*Caucasian (dark)*	Pune 18°52′N	T2DM	5	12	23	8	25	7
Rivera-Leon EA [49]	2015	Mexico	Mix	western of Mexico (Guadalajara 20°67′N)	T2DM	25	62	38	19	72	34
Vural HC [35]	2012	Turkey	*Caucasian*	Konya 37°86′N	T2DM	3	46	51	16	49	35
Wehr E [27]	2011	Austria	*Caucasian*	Graz 47°4′N	PCOS	72	238	226	23	65	49
Xu, J. R. [39]	2014	Chinese Han	Asian	Ningxia province 35–39°N	T2DM	176	24	1	172	47	0
		Chinese Hui			T2DM	134	17	3	99	16	0
Xu J.R. [38]	2012	China	Asian	Ningxia province 35–39°N	T2DM	182	19	0	188	25	1
Ye WZ [28]	2001	France	Caucasian	Paris 48°52′N	T2DM	49	136	120	23	66	54

**Table 4:** Characteristics of studies on VDR FokIrs2228570 (C > T) variant and Insulin resistance related diseases susceptibility

Author	Year	Country	Ethic	City latitude	Disease	Case			Control		
						TT	TC	CC	TT	TC	CC
Al-Daghri NM [18]	2012	Saudi	*Caucasian (dark)*	Riyadh 24°38′N	T2DM	213	133	22	129	111	19
Bagheri M [31]	2012	Iran	*Caucasian (dark)*	Urmia 37°33′N	PCOS	22	20	4	29	15	2
Bid HK [32]	2009	India	*Caucasian (dark)*	North Indian About 22–37°N	T2DM	2	60	38	1	79	80
Dasgupta S [48]	2015	India	*Caucasian (dark)*	Hyderabad 17°23′N	PCOS	8	87	155	9	88	152
Jia J [51]	2015	China	Asian	Nanjing 31°14′N	T2DM	120	336	212	408	973	579
					IFG	233	515	336	408	973	579
Jedrzejuk D [23]	2015	Poland	*Caucasian*	Wroclaw 51°1′N	PCOS	11	51	28	25	50	23
Mahmoudi T [24]	2009	Iran	*Caucasian (dark)*	Tehran 35°40′N	PCOS	12	67	83	7	59	96
Malecki MT [25]	2003	Poland	*Caucasian*	Krakow 50°08′N	T2DM	64	159	85	52	110	77
Mackawy A M [50]	2014	Eygpt	*Caucasian (dark)*	Zagazig 30°35′N	T2DM	34	40	66	5	11	44
					Mets	11	13	39	5	11	44
Shah DB [43]	2015	India	*Caucasian (dark)*	Telangana 17°49′N	T2DM	15	9	10	11	10	2
Schuch NJ [42]	2013	Brazil	Mix	São Paulo 23°33′N	Mets	40	47	13	35	57	8
Vedralová M [44]	2012	Czech Republic	*Caucasian*	Prague 50°05′N	T2DM	11	58	63	12	76	25
Wehr E [27]	2011	Austria	*Caucasian*	Graz 47°4′N	PCOS	82	241	215	22	60	53
Xia Z [36]	2014	China	Asian	Beijing 39°26′-41°03′N	T2DM	19	94	124	9	47	35
Yi Zhao [45]	2014	China	Asian	Yinchuan, Ningxia 38°2′N	MetS	75	184	132	80	207	112
Zhong X [30]	2015	China	Asian	Anhui Province 31°52′N	T2DM	44	114	46	18	58	40

### Association between VDR ApaI rs7975232 (G > T) variant and insulin resistance related diseases susceptibility

Fourteen studies (3212 cases and 3360 controls) examining the association between the VDR ApaI rs7975232 (G > T) variant and Insulin resistance related diseases susceptibility were included. Sub-group analysis (nine studies about T2DM and five studies about PCOS) was performed. All the original data were combined by means of the Random effect model. We found no association of the VDR ApaI rs7975232 (G > T) variant with Insulin resistance related diseases (OR, 1.08; 95% CI, 0.91–1.28; *P* = 0.37) in the recessive genetic model (G/G vs.G/T or T/T), dominant genetic model in the (G/G or G/T vs.T/T) (OR, 1.04; 95% CI, 0.89–1.21; *P* = 0.62) and G allele vs. T allele analysis (OR, 1.04; 95% CI, 0.95–1.1; *P* = 0.36). sub-group analysis indicated that there was no association between VDR ApaI rs7975232 (G > T)variant and T2DM, PCOS patients (Table 5). sub-group analysis by skin pigmentation and living latitude showed that ApaI rs7975232 (G > T) variant was associated with insulin resistance related diseases in Asians (GG/GT + TT) (OR, 1.62; 95% CI, 1.03–2.53; *P* = 0.04) and population who lived in middle latitude district (30–60°) (GG/GT + TT) (OR, 1.22; 95% CI, 1.04–1.43; *P* = 0.02). No publication bias was detected by either the funnel plot or Egger’s tests (*P* > 0.05, each comparison).

**Table 5:** Summary of meta-analysis

Comparison of outcome	No. of trials	No. of Case	No. of Control	Effect size (95% confidence intervals)	*P*	Test for heterogeneity	
						*I* ^2^ (%)	*P*
ApaI							
GG/GT + TT	14	3212	3360	1.08 [0.91, 1.28]	0.37	30	0.14
T2DM	9	2017	2555	1.00 [0.78, 1.28]	1	51	0.05
PCOS	5	1195	805	1.15 [0.88, 1.50]	0.31	0	0.47
GG + GT/TT	14	3212	3360	1.04 [0.89, 1.21]	0.62	38	0.08
T2DM	9	2017	2555	0.93 [0.79, 1.11]	0.44	17	0.29
PCOS	5	1195	805	1.15 [0.90, 1.45]	0.27	30	0.22
G allele	14	3212	3360	1.04 [0.95, 1.14]	0.36	26	0.18
T2DM	9	2017	2555	0.97 [0.85, 1.11]	0.7	42	0.1
PCOS	5	1195	805	1.11 [0.96, 1.27]	0.15	0	0.84
T allele	14	3212	3360	1.02 [0.91, 1.15]	0.7	56	0.0005
T2DM	9	2017	2555	1.03 [0.90, 1.18]	0.68	43	0.09
PCOS	5	1195	805	1.07 [0.83, 1.37]	0.62	70	0.01
Ethic							
GG/GT + TT	13	3087	3235	1.09 [0.91, 1.30]	0.34	34	0.11
Caucasian	5	1488	1929	1.20 [0.99, 1.45]	0.06	0	0.41
Caucasian (dark)	6	1091	1090	0.94 [0.64, 1.36]	0.73	52	0.07
Asian	2	508	216	1.24 [0.88, 1.76]	0.22	0	0.88
GG + GT/TT	13	3087	3235	1.08 [0.94, 1.24]	0.29	21	0.23
Caucasian	5	1488	1929	1.13 [0.87, 1.46]	0.36	49	0.1
Caucasian (dark)	6	1091	1090	0.97 [0.81, 1.15]	0.7	0	0.89
Asian	2	508	216	1.62 [1.03, 2.53]	0.04	0	0.35
G allele	13	3087	3235	1.06 [0.98, 1.16]	0.16	13	0.31
Caucasian	5	1488	1929	1.11 [0.98, 1.27]	0.06	0	0.51
Caucasian (dark)	6	1091	1090	0.96 [0.85, 1.09]	0.51	0	0.66
Asian	2	508	216	1.25 [0.99, 1.57]	0.1	17	0.3
T allele	13	3087	3235	1.01 [0.89, 1.14]	0.93	56	0.008
Caucasian	5	1488	1929	0.94 [0.80, 1.09]	0.4	42	0.14
Caucasian (dark)	6	1091	1090	1.16 [0.97, 1.38]	0.1	47	0.009
Asian	2	508	216	0.80 [0.64, 1.01]	0.06	0	0.51
Latitude							
GG/GT + TT	14	3212	3360	1.08 [0.91, 1.28]	0.37	30	0.14
Low (<30)	5	1136	834	0.86 [0.65, 1.14]	0.3	19	0.29
Middle (30–60)	9	2076	2526	1.22 [1.04, 1.43]	0.02	0	0.43
GG + GT/TT	14	3212	3360	1.04 [0.89, 1.21]	0.62	38	0.08
Low (<30)	5	1136	834	0.91 [0.73, 1.15]	0.44	17	0.31
Middle (30–60)	9	2076	2526	1.12 [0.92, 1.36]	0.27	42	0.08
G allele	14	3212	3360	1.04 [0.95, 1.14]	0.36	26	0.18
Low (<30)	5	1136	834	0.92 [0.80, 1.07]	0.27	10	0.35
Middle (30–60)	9	2076	2526	1.12 [1.01, 1.23]	0.02	0	0.44
T allele	14	3212	3360	1.02 [0.91, 1.15]	0.7	56	0.005
Low (<30)	5	1136	834	1.09 [0.94, 1.25]	0.26	10	0.35
Middle (30–60)	9	2076	2526	0.99 [0.84, 1.18]	0.95	66	0.003
BsmI							
AA/GA + GG	22	4294	4157	0.95 [0.78, 1.16]	0.64	41	0.02
T2DM	14	2802	3051	0.99 [0.75, 1.31]	0.93	55	0.007
PCOS	4	835	443	1.11 [0.77, 1.58]	0.58	0	0.61
MetS	4	657	663	0.72 [0.50, 1.05]	0.09	0	0.5
AA + GA/GG	22	4294	4157	1.06 [0.86, 1.31]	0.59	69	<0.00001
T2DM	14	2802	3051	1.19 [0.90, 1.57]	0.21	71	<0.001
PCOS	4	835	443	1.06 [0.79, 1.42]	0.7	19	0.29
MetS	4	657	663	0.62 [0.45, 0.86]	0.005	11	0.34
A allele	22	4294	4157	0.97 [0.83, 1.13]	0.67	72	<0.00001
T2DM	14	2802	3051	1.05 [0.85, 1.28]	0.67	76	<0.00001
PCOS	4	835	443	0.96 [0.79, 1.16]	0.65	12	0.33
MetS	4	657	663	0.71 [0.54, 0.93]	0.01	37	0.19
G allele	22	4294	4157	1.08 [0.89, 1.32]	0.42	83	<0.00001
T2DM	14	2802	3051	0.96 [0.78, 1.17]	0.67	76	<0.00001
PCOS	4	835	443	1.27 [0.67, 2.40]	0.73	91	0.00001
MetS	4	657	663	1.41 [1.07, 1.85]	0.01	37	0.19
Ethic							
AA/GA + GG	21	4194	4057	0.98 [0.80, 1.21]	0.87	40	0.03
Caucasian	7	1683	2121	1.01 [0.81, 1.26]	0.92	9	0.36
Caucasian (dark)	7	913	793	1.05 [0.82, 1.35]	0.69	0	0.82
Asian	7	1598	1143	0.90 [0.39, 2.08]	0.81	67	0.006
AA + GA/GG	21	4194	4057	1.10 [0.89, 1.36]	0.38	68	<0.00001
Caucasian	7	1683	2121	0.98 [0.82, 1.18]	0.84	25	0.24
Caucasian (dark)	7	913	793	1.50 [1.16, 1.93]	0.002	19	0.29
Asian	7	1598	1143	0.89 [0.49, 1.61]	0.69	80	<0.00001
A allele	21	4194	4057	1.02 [0.87, 1.19]	0.84	72	<0.00001
Caucasian	7	1683	2121	1.03 [0.86, 1.23]	0.75	59	0.02
Caucasian (dark)	7	913	793	1.23 [1.07, 1.42]	0.004	0	0.91
Asian	7	1598	1143	0.81 [0.49, 1.34]	0.42	86	<0.00001
G allele	21	4194	4057	1.06 [0.87, 1.29]	0.57	83	<0.00001
Caucasian	7	1683	2121	1.19 [0.85, 1.65]	0.32	89	<0.00001
Caucasian (dark)	7	913	793	0.81 [0.70, 0.94]	0.004	0	0.91
Asian	7	1598	1143	1.23 [0.74, 2.04]	0.42	86	<0.00001
Latitude							
AA/GA + GG	22	4294	4157	0.95 [0.78, 1.16]	0.64	41	0.02
Low (<30)	5	912	659	0.74 [0.52, 1.05]	0.09	39	0.16
Middle (30–60)	17	3382	3498	1.05 [0.83, 1.33]	0.68	37	0.06
AA + GA/GG	22	4294	4157	1.06 [0.86, 1.31]	0.59	69	<0.00001
Low (<30)	5	912	659	1.32 [0.73, 2.38]	0.35	70	0.009
Middle (30–60)	17	3382	3498	1.00 [0.81, 1.23]	0.97	61	0.0005
A allele	22	4294	4157	0.97 [0.83, 1.13]	0.67	72	<0.00001
Low (<30)	5	912	659	0.96 [0.64, 1.43]	0.83	80	0.0005
Middle (30–60)	17	3382	3498	0.97 [0.82, 1.15]	0.7	70	<0.00001
Gallele	22	4294	4157	1.08 [0.89, 1.32]	0.42	83	<0.00001
Low (<30)	5	912	659	1.04 [0.70, 1.56]	0.83	80	0.0005
Middle (30–60)	17	3382	3498	1.09 [0.87, 1.37]	0.44	84	<0.00001
TaqI							
TT/TC + CC	19	3533	4024	1.00 [0.82, 1.21]	0.96	60	0.004
T2DM	13	2305	3187	1.09 [0.84, 1.42]	0.51	60	0.003
PCOS	6	1228	837	0.86 [0.62, 1.20]	0.37	65	0.01
TT + TC/CC	19	3533	4024	0.88 [0.73, 1.06]	0.17	43	0.02
T2DM	13	2305	3187	0.92 [0.74, 1.14]	0.43	41	0.06
PCOS	6	1228	837	0.77 [0.51, 1.16]	0.22	52	0.06
T allele	19	3533	4024	0.89 [0.75, 1.06]	0.18	79	<0.0001
T2DM	13	2305	3187	1.01 [0.86, 1.18]	0.95	60	0.003
PCOS	6	1228	837	0.68 [0.48, 0.96]	0.03	84	<0.0001
C allele	19	3533	4024	1.13 [0.95, 1.34]	0.18	79	<0.0001
T2DM	13	2305	3187	0.99 [0.85, 1.17]	0.95	60	0.03
PCOS	6	1228	837	1.47 [1.03, 2.09]	0.03	84	0.00001
Ethic							
TT/TC + CC	17	3368	3859	0.93 [0.78, 1.12]	0.45	49	0.01
Caucasian	7	1653	2190	1.10 [0.90, 1.36]	0.35	38	0.14
Caucasian (dark)	7	1159	1121	0.75 [0.58, 0.97]	0.03	46	0.08
Asian	3	556	548	1.94 [0.32, 11.77]	0.47	0	0.44
TT + TC/CC	17	3368	3859	0.88 [0.72, 1.07]	0.2	48	0.01
Caucasian	7	1653	2190	1.12 [0.82, 1.53]	0.47	50	0.06
Caucasian (dark)	7	1159	1121	0.76 [0.57, 1.02]	0.07	39	0.13
Asian	3	556	548	0.67 [0.47, 0.96]	0.03	0	0.4
T allele	17	3368	3859	0.84 [0.71, 1.01]	0.06	78	<0.00001
Caucasian	7	1653	2190	0.94 [0.66, 1.33]	0.73	90	<0.00001
Caucasian (dark)	7	1159	1121	0.80 [0.68, 0.95]	0.01	41	0.12
Asian	3	556	548	0.73 [0.51, 1.04]	0.08	10	0.33
C allele	17	3368	3859	1.18 [0.99, 1.41]	0.06	78	<0.00001
Caucasian	7	1653	2190	1.06 [0.75, 1.51]	0.73	90	<0.00001
Caucasian (dark)	7	1159	1121	1.24 [1.05, 1.47]	0.01	42	0.11
Asian	3	556	548	1.37 [0.96, 1.94]	0.08	10	0.33
Latitude							
TT/TC + CC	18	3493	3984	0.95 [0.80, 1.12]	0.52	47	0.02
Low (<30)	5	934	896	0.86 [0.67, 1.09]	0.2	24	0.26
Middle (30–60)	13	2559	3088	1.00 [0.79, 1.25]	0.97	52	0.01
TT + TC/CC	18	3493	3984	0.87 [0.72, 1.05]	0.15	45	0.02
Low (<30)	5	934	896	0.88 [0.70, 1.12]	0.3	0	0.44
Middle (30–60)	13	2559	3088	0.87 [0.67, 1.13]	0.29	56	0.007
T allele	18	3493	3984	0.85 [0.72, 1.01]	0.06	77	<0.00001
Low (<30)	5	934	896	0.90 [0.78, 1.02]	0.11	0	0.69
Middle (30–60)	13	2559	3088	0.84 [0.66, 1.07]	0.15	83	<0.00001
C allele	18	3493	3984	1.17 [0.99, 1.39]	0.06	77	<0.00001
Low (<30)	5	934	896	1.11 [0.97, 1.27]	0.12	0	0.68
Middle (30–60)	13	2559	3088	1.19 [0.94, 1.51]	0.15	83	<0.00001
FokI							
CC/CT + TT	18	4992	6230	1.03 [0.82, 1.30]	0.79	80	<0.00001
T2DM	9	1086	690	1.10 [0.75, 1.60]	0.63	81	<0.00001
PCOS	5	631	559	1.20 [0.97, 1.48]	0.1	0	0.49
MetS	3	1084	1960	0.60 [0.16, 2.33]	0.46	93	<0.00001
CC + CT/TT	18	4992	6230	0.92 [0.72, 1.17]	0.49	74	<0.00001
T2DM	9	1086	690	1.02 [0.76, 1.37]	0.88	58	0.01
PCOS	5	631	559	1.29 [0.82, 2.03]	0.27	41	0.15
MetS	3	1084	1960	0.35 [0.10, 1.19]	0.09	93	<0.00001
C allele	18	4992	6230	0.99 [0.87, 1.12]	0.84	73	<0.00001
T2DM	9	1086	690	1.00 [0.79, 1.26]	0.99	81	<0.00001
PCOS	5	631	559	1.09 [0.85, 1.39]	0.49	54	0.07
MetS	3	1084	1960	0.75 [0.49, 1.14]	0.18	72	0.03
T allele	18	4992	6230	1.01 [0.89, 1.15]	0.85	73	<0.00001
T2DM	9	1086	690	1.00 [0.79, 1.26]	0.99	81	<0.00001
PCOS	5	631	559	0.92 [0.72, 1.17]	0.49	54	0.07
MetS	3	1084	1960	1.33 [0.87, 2.02]	0.19	73	0.03
Ethic							
CC/CT + TT	17	4892	6130	1.01 [0.80, 1.28]	0.92	80	<0.00001
Caucasian	4	1068	585	1.36 [0.77, 2.41]	0.29	83	0.0006
Caucasian (dark)	8	1240	1019	0.75 [0.41, 1.36]	0.35	86	<0.00001
Asian	5	2584	4526	1.13 [0.98, 1.30]	0.1	24	0.26
CC + CT/TT	17	4892	6130	0.91 [0.69, 1.20]	0.49	76	<0.00001
Caucasian	4	1068	585	1.25 [0.90, 1.74]	0.19	21	0.28
Caucasian (dark)	8	1240	1019	0.54 [0.26, 1.11]	0.09	82	<0.00001
Asian	5	2584	4526	1.13 [0.87, 1.47]	0.36	56	0.06
C allele	17	4892	6130	0.99 [0.86, 1.13]	0.83	74	<0.00001
Caucasian	4	1068	585	1.24 [0.92, 1.69]	0.16	74	0.01
Caucasian (dark)	8	1240	1019	0.77 [0.57, 1.04]	0.09	74	0.0003
Asian	5	2584	4526	1.06 [0.94, 1.18]	0.35	49	0.1
T allele	17	4892	6130	1.01 [0.89, 1.16]	0.84	74	<0.00001
Caucasian	4	1068	585	0.80 [0.59, 1.09]	0.16	74	0.01
Caucasian (dark)	8	1240	1019	1.29 [0.96, 1.74]	0.09	74	0.0003
Asian	5	2584	4526	0.95 [0.85, 1.06]	0.33	46	0.12
Latitude							
CC/CT + TT	18	4992	6230	1.03 [0.82, 1.30]	0.79	80	<0.00001
Low (<30)	5	852	791	1.00 [0.65, 1.52]	0.99	52	0.08
Middle (30–60)	13	4140	5439	1.03 [0.79, 1.36]	0.82	84	<0.00001
CC + CT/TT	18	4992	6230	0.92 [0.72, 1.17]	0.49	74	<0.00001
Low (<30)	5	852	791	0.78 [0.60, 1.01]	0.06	0	0.75
Middle (30–60)	13	4140	5439	0.94 [0.69, 1.26]	0.66	80	<0.00001
C allele	18	4992	6230	0.99 [0.87, 1.12]	0.84	73	<0.00001
Low (<30)	5	852	791	0.91 [0.74, 1.11]	0.36	33	0.2
Middle (30–60)	13	4140	5439	1.01 [0.86, 1.18]	0.93	78	<0.00001
T allele	18	4992	6230	1.01 [0.89, 1.15]	0.85	73	<0.00001
Low (<30)	5	852	791	1.10 [0.90, 1.35]	0.36	33	0.2
Middle (30–60)	13	4140	5439	0.99 [0.85, 1.16]	0.92	78	<0.00001

### Association between VDR BsmI rs1544410 (A > G) variant and insulin resistance related diseases susceptibility

Twenty-two studies (4294 cases and 4157 controls) in 17 papers examining the association between the VDR BsmI rs1544410 (A > G) variant and Insulin resistance related diseases susceptibility were included. Sub-group analysis (14 studies about T2DM, four studies about PCOS and four studies about Mets) was performed. All the original data were combined by means of the Random effect model. We found no association of the VDR BsmI rs1544410 (A > G)variant with Insulin resistance related diseases (OR, 0.95; 95% CI, 0.78–1.16; *P* = 0.64) in the recessive genetic model (A/A vs.A/G or G/G), dominant genetic model in th e (A/A or A/G vs. G/G) (OR, 1.06; 95% CI, 0.86–1.31; *P* = 0.59) and A allele vs. G allele analysis (OR, 0.97; 95% CI, 0.83–1.13; *P* = 0.67). sub-group analysis indicated that there was no association between BsmI rs1544410 (A > G) variant and T2DM, PCOS patients. However, significant association was found in MetS sub-group analysis G allele vs. A allele analysis (OR, 1.41; 95% CI, 1.07–1.85; *P* = 0.01) (Table 5). sub-group analysis by skin pigmentation and living latitude showed that VDR BsmI rs1544410 (A > G) variant was associated with insulin resistance related diseases in Caucasian (dark-pigmented) (AA + GA/GG) (OR, 1.50; 95% CI, 1.16–1.93; *P* = 0.002), (A allele) (OR, 1.23; 95% CI, 1.07–1.42; *P* = 0.004). No publication bias was detected by either the funnel plot or Egger’s tests (*P* > 0.05, each comparison).

### Association between VDR TaqI rs731236 (T/C) variant and insulin resistance related diseases susceptibility

Nineteen studies (3533 cases and 4024 controls) examining the association between the VDR Taq1rs731236 (T/C) variant and Insulin resistance related diseases susceptibility were included. Sub-group analysis (13 studies about T2DM, six studies about PCOS) was performed. All the original data were combined by means of the Random effect model. We found no association of the VDR TaqI rs731236 (T/C) variant with Insulin resistance related diseases (OR, 1.00; 95% CI, 0.82–1.21; *P* = 0.96) in the recessive genetic model (T/T vs.T/C or C/C), dominant genetic model in the (T/T or T/C vs. C/C) (OR, 0.88; 95% CI, 0.73–1.06; *P* = 0.17), T allele (OR, 0.89; 95% CI, 0.75–1.06; *P* = 0.18). Sub-group analysis indicated significant association between VDR Taq1rs731236 C allele and PCOS in C allele analysis (OR1.47; CI 1.03–2.09; *P* = 0.03) (Table 5). sub-group analysis by skin pigmentation and living latitude showed that VDR TaqI rs731236 (T/C) variant was associated with insulin resistance related diseases in Caucasian (dark-pigmented) (C allele) (OR, 1.24; 95% CI, 1.05–1.47; *P* = 0.01). No publication bias was detected by either the funnel plot or Egger’s tests (*P* > 0.05, each comparison).

### Association between VDR FokI rs2228570 (C > T) variant and insulin resistance related diseases susceptibility

Eighteen studies (4851 cases and 6174 controls) from 17 papers examining the association between the VDR FokIrs2228570 (C > T) variant and Insulin resistance related diseases susceptibility were included. Sub-group analysis (nine studies about T2DM, five studies about PCOS, three studies about MetS and one study about IFG) was performed. All the original data were combined by means of the Random effect model. We found no association of the VDR FokIrs2228570 (C > T)variant with Insulin resistance related diseases (OR, 1.00; 95% CI, 0.68–1.47; *P* = 0.99) in the recessive genetic model (C/C vs.C/T or T/T), dominant genetic model in the ((C/C or C/T vs. T/T) (OR, 0.86; 95% CI, 0.67–1.09; *P* = 0.21) and C allele vs. T allele analysis (OR, 0.96; 95% CI, 0.84–1.10; *P* = 0.53). sub-group analysis indicated that there was no association between FokIrs2228570 (C > T) variant and T2DM, PCOS and MetS patients (Table 5). sub-group analysis by skin pigmentation and living latitude showed that there were no association between VDR TaqI rs731236 (T/C) variant and insulin resistance related diseases in ethics with different skin pigment and in different latitudes. No publication bias was detected by either the funnel plot or Egger’s tests (*P* > 0.05, each comparison).

## Discussion

VDR, which is considered as a pleiotropic gene, is a transcription factor that mediates the action of vitamin D3 by controlling the expression of hormone sensitive genes such as Calmodulin-Dependent Kinase (CaMKs), and CaMKs stimulates VDR-Mediated transcription by phosphorylation levels of VDR [46]. Recent research found that deletion of macrophage VDR promotes insulin resistance and monocyte cholesterol transport to accelerate atherosclerosis in mice [47] which suggested that VDR dysfunction might result in insulin resistance. The association between VDR polymorphisms and insulin resistance related diseases including T2DM, PCOS and Mets has been extensively researched, but the results obtained so far are conflictive, and the role of VDR polymorphisms remains unclear. The reasons for this disparity may be small sample sizes, low statistical power, differences in ethnicities, extensive geographic variations, and interactions with other genetic or environmental factors. Therefore, in order to overcome the limitations of individual studies, we performed a meta-analysis. Meta-analysis increases statistical power and resolution by pooling the results of independent analyses. In this meta-analysis, we combined data from published case–control studies to evaluate the genetic associations of TaqI, BsmI, ApaI and FokI polymorphisms with these insulin resistance diseases.

To the best of our knowledge, this is the first meta-analysis which takes into account the interaction of individual VDR polymorphisms with in insulin resistance diseases. This meta-analysis, which included a total of 28 articles, examined the associations among four studied polymorphisms in the VDR ApaI variant, VDR BsmI variant, VDR Taq1 variant and VDR FokI variant and insulin resistance related diseases. The results indicated that VDR ApaI variant, VDR BsmI variant and VDR FokI variant were not conspicuous risk factors for insulin resistance related diseases. The result provided no evidence of the association between VDR variant and Insulin resistance related diseases. Yet the results were different when the researches were grouping by skin pigment and living latitude. Sub-group analysis suggested that the association between insulin resistance related diseases and VDR ApaI, BsmI, FokI variant was obvious in dark-pigmented Caucasian population and Asians.

However, to make conclusive estimates, many factors should be considered. In complex diseases such as T2DM, complex interactions between genetic and environmental factors have differential effects on disease susceptibility. Further characterization of VDR, in addition to traditional and related risk factors may facilitate early identification of patients at high risk for T2DM, and then elucidate new approaches for prevention and treatment. However, several limitations of the meta-analysis should be addressed. First, lack of the original data of the reviewed studies limited our further evaluation of potential interactions, because the interactions between and even different polymorphic loci of the same gene may influence the risk. Second, our results were based on unadjusted published estimates, and hence, we were unable to adjust them by possible confounders, for example Vitamin D level, and diet did not take into consider. Third, the number of articles and cases taking in this research is relatively small. In order to provide a more precise estimation on the basis of adjustment for confounders, more well-designed studies should be taking into account. Additionally, current evidence from prospective studies on the association between vitamin D gene polymorphism and risk of insulin resistance related diseases was limited by the use of vitamin D gene polymorphism or a single measurement of 25(OH)D concentrations. A single baseline measure of dietary vitamin D may not be able to take into account the within-individual variations of vitamin D levels across seasons or geographical location, as evident in sub-group analysis. Studies are, therefore, needed with geographical location and dietary vitamin D levels to adjust for its variability while quantifying the associations.

## Conclusion

In summary, this meta-analysis provided evidence of the association between VDR BsmI variant and MetS and supporting that VDR BsmI variant G allele might be a susceptibility marker of MetS. TaqI variant was associated with PCOS for C allele and supporting that VDR TaqI variant C allele might be a susceptibility marker of PCOS. No significant association was found in the rest gene polymorphisms and these diseases related with insulin resistance diseases. The relationship of VDR gene polymorphism was more important with PCOS and MetS than T2DM. However, sub-group analysis showed VDR ApaI variant was associated with insulin resistance related diseases in Asians, VDR BsmI and VDR TaqI variant was associated with insulin resistance related diseases in Caucasian (dark-pigmented).The results suggested that the association between insulin resistance related diseases and VDR ApaI, BsmI, FokI variant was more obvious in dark-pigmented Caucasians and Asians but not in Caucasian with white skin.

## Abbreviations

MetS: Metabolic syndrome
PCOS: Polycystic ovarian syndrome
T2DM: Type 2 diabetes
VDR: Vitamin D receptor
